# Effect of pre-operative administration of single-dose of gabapentin on postop surgical pain in patients undergoing sternotomy for CABG

**DOI:** 10.12669/pjms.38.6.5518

**Published:** 2022

**Authors:** Kashaf Ilyas, Rukhsana Latif, Zainab Hafeez

**Affiliations:** 1 Dr. Kashaf Ilyas, MBBS, Department of Cardiology, Ch. Pervaiz Elahi Institute of Cardiology, Multan, Pakistan; 2 Dr. Rukhsana Latif, MBBS, Department of Cardiology, Ch. Pervaiz Elahi Institute of Cardiology, Multan, Pakistan; 3 Dr. Zainab Hafeez, MBBS, Department of Cardiology, Ch. Pervaiz Elahi Institute of Cardiology, Multan, Pakistan

**Keywords:** Gabapentin, CABG, Post-surgical pain, Sternotomy, Outcomes

## Abstract

**Objective:**

To investigate the efficacy of perioperative administration of a single dose of gabapentin on postoperative pain in patients undergoing sternotomy for CABG.

**Methods:**

A cross-sectional analytical study was conducted at the cardiology ward of Ch. Pervaiz Elahi Institute of Cardiology, Multan, form 9^th^ June 2020to 9^th^ June 2021. The participants were consecutively enrolled in the Gabapentin group, who received 1200 mg of Gabapentin two hours before the surgery for the two days, and the placebo group. The outcomes measurement involved the need for fentanyl intake in the first two postoperative days; pain at rest and in the moment in the first three postoperative days; postsurgical sleep scores; and patients reported quality of recovery.

**Results:**

A total of 50 patients were enrolled in the study, 25 in each group. No significant difference was found between the two groups in terms of fentanyl intake, sleep scores, visual analog pain score, and the need for any adjunctive pain medication (p>0.05). Similarly, no significant difference was found in terms of occurrence of side-effects and quality of recovery (p>0.05).

**Conclusion:**

Pre-operative administration of a single dose of Gabapentin doesn’t significantly influence the post-operative pain, opioid consumption, sleep quality, or quality of recovery in patients undergoing cardiac surgery.

## INTRODUCTION

Sternotomy, either for valvular surgery or coronary artery bypass graft (CABG), is mostly followed by significant pain at incisional as well as excisional sites, including chest tube insertion region, from pericardiotomy, and at saphenous vein excision point.^1^,^2^ This post-surgical pain continues to pose prevention and management challenges despite achieving good surgical results. Consequently, unmanaged post-sternotomy pain contributes to a high mortality rate, prolonged hospital stay, and worsen clinical outcomes.^3^,^4^ Post-surgical pain experience after non-cardiac surgeries is usually managed through various analgesics, but they are found to have limited efficacy in cardiac surgeries since their side effects can impact the outcomes of surgical intervention.

Similar to non-steroidal anti-inflammatory drugs, opioids, prime post-cardiac surgery pain management drugs, also have a range of side effects and persistent local anesthesia infusion may end up in tissue infection and necrosis. Acute post-surgical pain may have an underlying neuropathic phenomenon that involves various mechanisms resulting in central and peripheral neural sensations.

Gabapentin, an anti-epileptic drug, reduces neuropathic and nociceptive pain by binding to the α_2_δ subunit of voltage-regulated calcium channels.^5^ Several studies and meta-analyses have been conducted to test the efficacy of gabapentin in controlling perioperative pain.^6^,^7^ It has been analyzed that gabapentin is relatively safe and has minimal drug interactions in cases of cardiac surgery.^6^,^8^ However, the effect of the drug on post-cardiac surgery pain is not extensively investigated. Therefore, the present placebo-controlled study aimed to investigate the efficacy of perioperative administration of a single dose of Gabapentin on postoperative pain in patients undergoing sternotomy for CABG. Besides, the study also investigated the effects of the drug on other related endpoints: Post-surgical sleep and patient-reported post-surgical quality of recovery. The study will assist in finding an effective treatment for post-cardiac surgery pain which will ultimately reduce the associated mortality rate.

## METHODS

A placebo-controlled cross-sectional study was conducted from 9^th^ June 2020to 9^th^ June 2021 at the Cardiology Department of Ch. Pervaiz Elahi Institute of Cardiology, Multan. A total of 50 patients aged between 18 to 60 years and undergoing sternotomy for CABG were consecutively included in the study. Patients allergic to Gabapentin; those with a history of chronic pain disorder or substance abuse; renal or liver insufficiency; left ventricular ejection fraction< 40%; emergency surgery; myocardial infarction in last two days, and who utilized ketamine for anesthesia induction were excluded. Similarly, patients with intraoperative CBP>3hrs.; post-operative symptomatic dysrhythmias; 35.9 C>body temperature <38.5 C; and urine output <30ml/hour. for consecutive four hours. were dropped from the study. Patients were informed of the study’s aims and written consent was sought. Ethical approval was obtained Ref # 28/115; dated June 2, 2020 from the ethical committee of the hospital. On the surgery day, the initial 25 participants were assigned to the gabapentin group who were administered a single dose of 1200mg gabapentin and the later 25 were administered placebo capsules, apparently similar to gabapentin. Following the surgery, all patients were kept on a fentanyl patient-controlled analgesia pump (PCA) as the initial postsurgical pain management medication and all the secondary pain-relieving drugs were administered through PCA before extubation. However, PCA was replaced by oral opioids 48 hours postoperatively. Besides gabapentin, all participants were also given 1g paracetamol four times a day. The standard protocol of the surgery was followed by a trained surgeon for every patient and no limitation was reported in any patient in terms of site of graft placement. All the medical staff involved in the study was kept blind to the division of patients into two groups.

The two groups were primarily compared in terms of the amount of fentanyl consumption in the initial two days postoperatively. Additionally, a visual analog scale (VAS) (0= no pain at all to 10= severe pain) was utilized to measure pain at rest and in movement at 12, 24, 48, and 72 hours postoperatively; the amount of additional pain medication (pethidine, tramadol, or any other nonsteroidal anti-inflammatory drug) was assessed; patient assessed sleep quality at postoperative 2^nd^ and 3^rd^ night were noted; gabapentin-related side-effects, particularly sedation, diarrhea, arrhythmias, and dizziness. Moreover, since gabapentin is reported to be an anxiolytic drug and contributes to functional recovery, patients were investigated about their satisfaction with pain management through the quality of recovery (QoR) questionnaire. The QoR score offers a reliable method of evaluating the post-surgical recovery of patients and is suitable for various surgical interventions.^9^ Patients scored their sleep quality at 24 and 48 hours postoperatively on a scale of 0 to 5 (0=most uncomfortable sleep, 5= very comfortable sleep). Whereas, sedation scores were allotted by nursing staff at 1^st^ and 2^nd^ day on a scale of 0 to 3, such that 0=patient completely conscious; 1= patient under sedation but responding to verbal directions; 2= patient under sedation but responding to tactile stimuli, and 3=patient under sedation and responding to only pain perceptions. Patients themselves rated their state of dizziness on a scale of 1 to 5 (1= complete absence of dizziness, 5= severe dizziness). Patients were also assessed of their demographic data; time on cardiopulmonary bypass (CBP); time under anesthesia; magnesium intake rate, and time to extubation. Magnesium, an N-methyl-D-aspartate receptor antagonist, has been reported to reduce the opioid need in the experimental animal model and it is administered to cardiac surgery patients to prevent arrhythmia.

SPSS (version 18.0) was used for statistical analysis. Continuous variables were presented as mean along with standard deviation (SD) while categorical were presented as percentages. The significance of the difference in qualitative and quantitative data between the two groups was assessed through chi-square and student’s t-test. However, since the data about fentanyl usage and VAS score didn’t follow a normal distribution, it was compared by Mann-Whitney U-test. A P-value less than 0.05 was considered statistically significant for any variable.

## RESULTS

Out of 50 enrolled patients, three were excluded from the gabapentin group and one from the placebo group due to certain postoperative complications resulting in a total of 46, 22 in the gabapentin group and 21 in the placebo group. No significant difference was found between the two groups in terms of demographics and clinical data except for the higher number of patients with diabetes mellitus (45.4%) and those who received magnesium (40.9%) in the gabapentin group than those in the placebo group, 12.5%, and 16.6% respectively (Table-I). The patients in the two groups reported almost similar results in terms of total fentanyl intake in the first two postoperative days; usage of additional pain medication sleep score; the number of anti-emetics; and self-perceived quality of recovery (Table-II). Similarly, the mean VAS score didn’t vary significantly between the two groups at 12, 24, 48, and 72 hours (Table-III). The side-effects, arrhythmias, and dizziness was also not significantly different between the groups (Table IV).

**Table I:** Demographic and operative data of participants (N=46).

Variables	Gabapentin (N=22)	Placebo (N=24)	P-value
Gender, M/F	17/5	21/3	0.8
Age, years	59.8±9.7	55.6± 7.1	0.63
BMI	25.2±18	27.1± 21	0.41
Diabetes, n	10 (45.4%)	3 (12. 5%)	0.03
Hypertension, n	13 (59%)	15 (62.5%)	0.7
Time under anesthesia	266± 60	272± 59	0.52
Time on CPB, (min ± range)	82.5 ± 26	87 ± 29	0.9
Time to extubation, (min ± range)	573 ± 241	549± 236	0.71
Magnesium is given, n	9 (40.9%)	4 (16.6%)	0.02

**Table II:** Postoperative outcome of the study group (N=46).

Outcome	Gabapentin (N=22)	Placebo (N=24)	P-value
Total fentanyl intake (ug)	1349±989	1509±1014	0.46
QoR questionnaire score	14.9 ±1.9	13.8±2.1	0.07
Number of anti-emetics used per patient	2.1±2.1	3.0±2.7	0.24
Additional pain medications intake	0 (0%)	3 (12.5%)	0.07
Sleep score at 24 hrs.	2.2± 0.9	2.0 ±1.4	0.75
Sleep score at 48 hrs.	2.9 ± 0.9	2.9 ± 0.9	1.0

**Table III:** Pain score at rest and movement at specific study period (N=46).

Time (post-operative hrs.)	Activity level	Gabapentin (N=22)	Placebo (N=24)	P-value
12	Rest	4 (0-9)	2 (0-5)	0.17
	Movement	4 (0-8)	5 (0-8)	0.52
24	Rest	3 (0-7)	3 (0-6)	0.3
	Movement	4 (0-9)	6 (0-9)	0.4
48	Rest	1 (0-8)	2 (0-5)	0.08
	Movement	4 (0-7)	4 (0-9)	0.75
72	Rest	1 (0-6)	1 (0-4)	0.53
	Movement	4.5 (0-8)	3 (0-7)	0.31

**Table IV:** Side-effects of Medication.

Side-effects	Gabapentin (N=22)	Placebo (N=24)	P-value
Arrhythmia, n	2 (9%)	6 (25%)	0.41
Dizziness score	0.79±1.5	0.51± 1.1	0.39
Sedation score at Day-1	0.89±0.58	0.75 ±0.73	0.76
Sedation score at Day-2	0.2±0.61	0.91±0.45	0.08

## DISCUSSION

The study has concluded that preoperative intake of a single dose of gabapentin doesn’t significantly affect opioid utilization following cardiac surgery. Additionally, mean VAS pain scores were almost similar in the study and placebo groups both at rest and in the movement. Similarly, no significant difference was found in sleep quality on their self-evaluated quality of recovery. However, gabapentin didn’t produce considerable side-effects as both the groups had no significant difference in terms of incidence of evaluated side-effects.

Gabapentin, structurally similar to gamma-aminobutyric acid, is an antiepileptic drug. While certain studies recommend multiple doses of gabapentin, a considerable number of meta-analysis and clinical trials suggest that the intake of a single dose of gabapentin is as effective as other perioperative procedures employed for pain management.^6^,^10^ The gabapentin achieves its peak plasma level within two to three hours following the intake which afterward gets eliminated through urinary output, with the clearance rate associated with creatinine clearance.^11^,^12^ The dose considered for the present study was within the bracket of a single dose and is in agreement with the dosage utilized in several dose-finding studies and clinical trials conducted to evaluate the effect of gabapentin administration on postoperative pain.^8^,^13^,^14^

In the last few decades, cardiac surgery has witnessed major improvements in certain technical aspects, including surgical techniques, protection of myocardium on CPB, anesthetic monitoring, and medications. However, postoperative pain management is still greatly dependent on opioid management. As the age of patient advances, comorbid disorders become prevalent; hence multiple therapies are often required to preserve cognitive functions. Considering other perioperative trials in non-cardiac surgeries, it was hypothesized that preoperative intake of gabapentin would reduce the opioid utilization postoperatively in cardiac surgery patients and also decrease the incidence of opioid-associated side-effects including vomiting, constipation, sedation, and respiratory depression that may lead to the mechanical ventilation. Several studies suggest the occurrence of acute neuropathic pain following surgical procedures and it is common to experience dysaesthesia in cardiac surgery patients that are usually unrelieved by traditional opioid analgesia.^15^ Gabapentin has been reported effective by certain studies in managing this type of pain and being a component of postsurgical multimodal analgesia, it can be a reliable choice as a co-analgesia where multiple nociceptor mechanisms occur.^16^ However, our study concluded no significant benefits of gabapentin in reducing postoperative pain, sleep improvement, decrease in opioid intake, quality of recovery, and reduction of opioid-associated side-effects. The same results have also been recently reported by some related studies.^17^-^19^ However, contrasting results are also reported which declares single-dose administration of gabapentin as a useful key to postoperative pain management.^20^ Therefore, further studies are suggested to validate the efficacy and the optimum dosage of the evaluated drug.

### Limitations of the study

It includes smaller size and short follow-up period couldn’t assess the role of gabapentin in chronic postoperative pain management.

## CONCLUSION

Pre-operative administration of a single dose of gabapentin doesn’t significantly influence the post-operative pain, opioid consumption, sleep quality, or quality of recovery in patients undergoing cardiac surgery.

### Authors Contribution:

**KI, ZH:** Conceived, designed and did statistical analysis & editing of manuscript.

**RL, ZH:** Did data collection and manuscript writing.

**ZH, KI:** Did review and final approval of manuscript.

**RL, KI:** Responsible & accountable for accuracy of the work.
